# Characterization of the protein fraction of the extracellular polymeric substances of three anaerobic granular sludges

**DOI:** 10.1186/s13568-019-0746-0

**Published:** 2019-02-07

**Authors:** Charles-David Dubé, Serge R. Guiot

**Affiliations:** 1 Anaerobic Bioprocesses Group, Energy, Mining and Environment Research Centre, National Research Council Canada, 6100 Royalmount Avenue, Montreal, H4P 2R2 Canada; 2 Department of Microbiology, Infectiology and Immunology, Université de Montréal, 2900 Boul. Édouard-Montpetit, Montreal, H3T 1J4 Canada

**Keywords:** Anaerobic granule, Extracellular polymeric substances, Humic substances, Proteins, Mass spectrometry

## Abstract

Extracellular polymeric substances (EPS) play major roles in the efficacy of biofilms such as anaerobic granules, ranging from structural stability to more specific functions. The EPS of three granular anaerobic sludges of different origins were studied and compared. Particularly, the peptides from the protein fraction were identified by mass spectrometry. *Desulfoglaeba* and *Treponema* bacterial genera and *Methanosaeta* and *Methanobacterium* archaeal genera were prominent in all three sludges. *Methanosaeta concilii* proteins were the most represented in EPS of all three sludges studied. Principally, four proteins found in the three sludges, the S-layer protein, the CO-methylating acetyl-CoA synthase, an ABC transporter substrate-binding protein and the methyl-coenzyme M reductase, were expressed by *Methanosaeta concilii*. Mainly catabolic enzymes were found from the 45 proteins identified in the protein fraction of EPS. This suggests that EPS may have a role in allowing extracellular catabolic reactions.

**Electronic supplementary material:**

The online version of this article (10.1186/s13568-019-0746-0) contains supplementary material, which is available to authorized users.

## Introduction

The biofilm is the best-known community lifestyle for microorganisms. Extracellular polymeric substances (EPS) embed biofilm cells, thus play an important role in the biofilm development and cohesion. The EPS macromolecules are either excreted by microorganisms, produced from cell lysis, or adsorbed from the external environment (e.g. wastewater) (Sheng et al. 2010). EPS include mostly proteins, carbohydrates, humic substances (HS) and nucleic acids. The question of which microbial species participates most in mixed species biofilm matrix construction remains, in most cases, unclear.

Anaerobic sludge granules are small spherical biofilms, which contain the different microbial groups typically found in anaerobic digestion consortia. Each group performs specialized metabolic functions sequentially leading to the transformation of the primary substrate ultimately into methane and CO_2_. Compared to aerobic granules, anaerobic granules have a longer start-up period (2–8 months) but they have a much stronger stability (Ding et al. 2015; Liu et al. 2004). However, for both granules types, their EPS content has been found much higher than in any other biofilm types (Tay et al. 2001). EPS are known to be important in the maintenance of granule stability and cohesion (Ding et al. 2015; MacLeod et al. 1995). The EPS matrix can also trap extracellular catalytic enzymes and keep them in close proximity to the cells (Zhang et al. 2015). EPS can protect the cells against oxygen and toxic compounds, allow for sorption of organic and inorganic compounds, act as an electron donor and acceptor, and facilitate communication among the cells through biochemical signals as well as gene exchange (Flemming and Wingender 2010).

In most anaerobic sludge systems, the largest fraction of EPS extracts are proteins, which significantly contribute to the granule formation (Zhu et al. 2015). EPS extracts from aerobic and anaerobic granules have quite the same characteristics; they both have proteins as the main EPS fraction and a protein/carbohydrate (PN/PS) ratio in EPS from 1 up to 12. However, what seems to differentiate the anaerobic granules from the aerobic ones is the presence of an important fraction of HS (Ding et al. 2015). Proteins from aerobic sludge EPS had previously been studied by sodium dodecyl sulfate polyacrylamide gel electrophoresis (SDS-PAGE) and mass spectrometry (Park et al. 2008; Zhang et al. 2007), but never those from anaerobic sludge. The identification of these proteins and their origin could help us to clarify the hypothetical roles of EPS and better understand the microbial ecology of anaerobic granular biofilms and the adhesion mechanisms behind granules formation in upflow anaerobic sludge blanket (UASB) reactors.

This study focuses on comparison of three anaerobic granular sludges treating different industrial effluents. The first objective was to characterize their populations in order to comprehensively assess species ubiquity and specificity among the different granule types. The second objective was to extract and identify EPS proteins of each granule type and correlate these findings to the microbial species characterization in order to better understand the roles that EPS and these species could play within the anaerobic granules in relation to the different feed substrates.

## Materials and methods

### Sources of sludge and preparation

The anaerobic granular sludge samples used in this study came from three large-scale reactors of the UASB type, treating industrial wastewater. The three companies were Agropur, a cheese factory, Lassonde, a fruit juice factory, and Tembec, a pulp and paper mill, respectively (Table 1). Sludge was stored at 4 °C until experiments. Before experiments, sludge was rinsed with anoxic carbonate buffer of pH 7.0 containing (g/L) NaHCO_3_ (10), NH_4_Cl (0.5), KH_2_PO_4_ (0.3) and K_2_HPO_4_ (0.4) (Dolfing and Mulder 1985), to remove free cells and debris. To determine the dry weight (DW) and the volatile dry weight (VDW) of the sludge, samples were heated overnight at 105 °C and 1 h at 600 °C, respectively (Eaton et al. 2005).

**Table 1:** Feeding characterizations and operational conditions of the reactors

	Agropur	Lassonde	Tembec
Effluent treated	Cheese factory	Fruit juice factory	Pulp and paper mill
Digester volume capacity (m^3^)	550	400	2000
Retention time (average) (h)	12	24	6
pH in digester	6.8–6.9	6.8–7.2	7.1–7.2
Total COD (wastewater in) (mg/L)	2400	4500–7000	6000–8000
Soluble COD (wastewater in) (mg/L)	1400	4500–6000	6000–8000
Total COD (effluent) (mg/L)	800	300–500	1000–2000
Soluble COD (effluent) (mg/L)	≤ 50	200	1000–2000
Ammonia (effluent) (mg/L)	90	≤ 1	20–30
Granules (square = 1 mm^2^)			

### Microbial community analysis by high throughput screening (HTS)

Total genomic DNA was extracted from the sludges samples using the PowerSoil™ DNA isolation kit (Mobio Laboratories, Carlsbad, CA) according to the manufacturer**’**s instructions. Bacterial 16S rRNA genes were amplified using the set of primers E786F (5′ GATTAGATACCCTGGTAG 3′) and U926R (5′ CCGTCAATTCCTTTRAGTTT 3′) (El Fantroussi et al. 1998). Archaeal 16S rRNA genes were amplified using the set of primers 958arcF (5′ AATTGGANTCAACGCCGG 3′) and an equimolar mix of 1048arcR-major (5′ CGRCGGCCATGCACCWC 3′) and 1048arcR-minor (5′ CGRCRGCCATGYACCWC 3′) (Hadjeb and Berkowitz 1996). A sample-specific multiplex identifier was added to each forward primer and an Ion Torrent adapter (Thermo Fisher Scientific, Waltham, MA) was added to each primer. The polymerase chain reaction (PCR) amplification reactions were performed as described previously (Hussain et al. 2014) using the rTaq DNA polymerase (GE Healthcare, Life Sciences Piscataway, NJ). The PCR products were purified and quantified using a QIAquick gel extraction kit (Qiagen, Valencia, CA) and a Quant-iT PicoGreen double-stranded DNA quantitation kit (Life Technologies Inc., Carlsbad, CA), respectively, according to the manufacturer’s instructions. The pooled bacterial and archaeal 16S rRNA gene amplicons were then sequenced using Ion Torrent sequencing platform with a 314 chip, according to the manufacturer’s instructions. For sequencing analysis, bacterial and archaeal 16S rRNA gene sequences generated were analyzed using the ribosomal database project (RDP) classifier (Schmidt 1986) using bootstrap confidence cutoffs of 50%, as recommended by the RDP classifier for short sequences (less than 250 bp) (Juteau et al. 1995). Sequences that were shorter than 75 bp and sequences with unidentified bases (N) were removed from the analysis. Sequences have been deposited in NCBI database (accession number PRJNA498054).

### EPS extraction

Sonication (Sonics Vibra Cell VC130, probe CV18 3987, Sonics & Materials Inc., Newtown, CT) was used for granules disruption and EPS extraction. Various times of sonication (2, 4, 8 and 12 min) at 30 W (1 W/mL) were compared. After 2 and 4 min, a large number of intact granules were still recovered, meaning that extraction was too short to break down all granules. The amount of EPS extracted tended to increase with the sonication duration. However this tendency faded between 8 and 12 min, and after 8 min of sonication, no intact granules could be recovered. We thus considered that the optimal sonication time to obtain EPS was 8 min and longer sonication times would likely affect the cell integrity. The PN/PS ratio had a slight tendency to decrease with increasing time of granule exposure to sonication (variance of 27% between the four times tested).

After rinsing and removal of the water excess, 15 g of wet granules were added to 15 mL of carbonate buffers and then sonicated at 30 W (1 W/mL) for 8 min. Sonication was performed on ice with a 2 min interval between each 2 min of sonication, to prevent overheating. The sonicated samples were then centrifuged at 15,000×*g*, 3 times 15 min (Sorvall RC6Plus, Thermo Electron Corporation, Waltham, Massachusetts). The supernatant containing EPS were frozen at − 20 °C until further characterization or directly used for precipitation steps (see SDS-PAGE section) and cell lysis detection. Each EPS extraction, cell lysis detection and characterization was done in triplicate for each sludge. The triplicates were pooled together prior the gel electrophoresis and mass spectrometry.

### Cell lysis detection

Sonication could damage the cell membrane and induce cell lysis, depending on the strength and duration of sonication (Picard et al. 1992). Glucose-6-phosphate dehydrogenase (G6PDH) (G8404, Millipore Sigma, ON, CA) activity was measured on EPS extract to evaluate the cell lysis during the sonication (Monique et al. 2008). 800 µL of enzymatic substrate solution (0.2 M Tris-HCl, 0.2 M 2 mercaptoethanol, 0.5 mM NAD, 10 mM d-glucose-6-phosphate, pH 8.5) were added to 200 µL of each extract. The absorbance was measured at 340 nm at room temperature every 35 s using UV1 (Thermo Spectronic) for 10 min to measure NADH production. 1U of G6PDH gives 1 nmol of NADH produced per minute, and it is equivalent to approximately 5 × 10^6^ lysed cells, or 20 ng of dry lysed cells (Ras et al. 2011).

### Characterization of EPS

DW and VDW of EPS extracts were determined as mentioned above to calculate yields of extraction. Protein, carbohydrate, humic substance and nucleic acid fractions were determined by colorimetric methods. All measurements were done using a DR 3900 spectrophotometer (Hach, London, ON, CA). Carbohydrates were determined following the Dubois’ protocol (Dubois et al. 1956) and nucleic acids, the Burton’s protocol (Burton 1956). Proteins and humic substances were determined using Lowry-based Frølund’s method (Frølund et al. 1995; Lowry et al. 1951), modified as detailed in the Additional file 1: Figures S1, S2.

### Separation using gel electrophoresis

Prior separation on sodium dodecyl sulfate polyacrylamide gel electrophoresis (SDS-PAGE), sequential precipitations of EPS extracts were done to roughly separate the protein from the HS fraction, which precipitates at lower salt concentration (Park and Helm 2008). Ammonium sulfate was stepwise added from 0.2 to 0.6 g/mL (+ 0.1 g/mL at each step), incubated for at least 6 h at 4 °C and then centrifuged at 20,000×*g*, 30 min at 4 °C. The centrifugation pellets were resuspended in 1.5 mL of carbonate buffers. SDS-PAGE (Laemmli 1970) were done on 4–12% bis–tris polyacrylamide gels and coloured with Coomassie blue (Bio-rad, Mississauga, ON, CA). During the EPS precipitation steps with ammonium sulfate, humic substances tended to precipitate in fractions with between 0 and 0.3 g (NH_4_)_2_SO_4_/mL while proteins predominated in the other fractions, with 0.4 to 0.6 g (NH_4_)_2_SO_4_/mL. Bands of proteins appeared in the last three fractions (i.e. with 0.4 to 0.6 g (NH_4_)_2_SO_4_/mL). Those three fractions were pooled and passed on the gels.

### Mass spectrometry (MS)

**Fig. 1:**
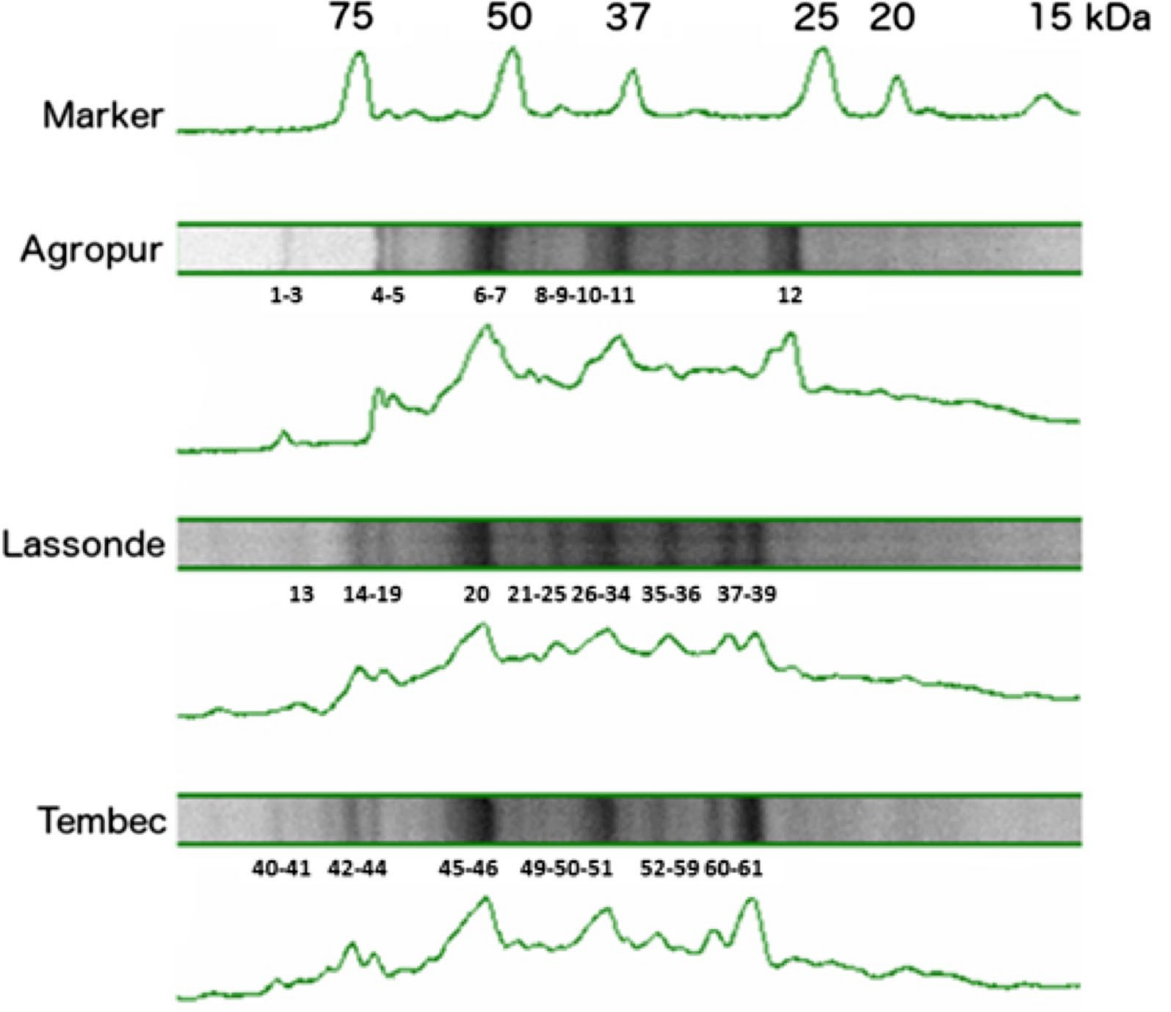
Electrophoresis of precipitated proteins from Agropur, Lassonde and Tembec sludges. *Migration is from left to right*

The more representative protein bands (shown on Fig. 1) were cut from the gel for each sample (about 8 bands per sample). Bands were detained with water/sodium bicarbonate buffer and acetonitrile. The protein fragments were reduced with dithiothreitol (DTT) and alkylated with iodoacetamide prior to in-gel digestion with trypsin. The tryptic peptides were eluted from the gel with acetonitrile containing 0.1% of tri-fluoro-acetic acid and then separated on an Agilent Nanopump using a C18 ZORBAX trap and a SB-C18 ZORBAX 300 reversed phase column (150 mm × 75 µm, 3.5 µm particle size) (Agilent Technologies Inc., Santa Clara, CA). All mass spectra were recorded on a hybrid linear ion trap-triple quadrupole mass spectrometer (Q-Trap, Applied Biosystems, MDS SCIEX Instruments, Concord, ON, CA) equipped with a nano-electrospray ionization source. The accumulation of tandem mass spectrometry (MS/MS) data was performed with the Analyst Software, version 1.4 (Applied Biosystems, MDS SCIEX). MASCOT (Matrix Science, London, UK) was used to create peak lists from MS to MS/MS raw data. Only individual ion scores that indicate identity or extensive homology (p < 0.05) and only proteins with at least one significant sequence were kept for interpretation.

## Results

### Microbial populations

The molecular-based profiles of the microbial communities were first determined in the three anaerobic sludges under investigation (i.e. Agropur, cheese factory; Lassonde, juice industry; Tembec, paper mill), using high throughput screening (HTS). Those three sludges were chosen because they treat wastewaters of various origins, that we know could have a great impact on microbial populations and EPS composition (Liu et al. 2004; Sheng et al. 2010). Results are shown in Table 2. Some genera found at high levels in some sludge were totally absent in others. This is the case for the bacterial genera *Mesotoga* and *Syntrophobacter* (absent in the Tembec sludge), *Propioniciclava* (absent in the Agropur and Tembec sludges), *Desulfovirga* (absent in the Lassonde sludge). On the other hand, bacterial genera such as *Desulfoglaeba* and *Treponema* were relatively abundant in all sludges. Yet a large percentage of bacterial genera remained unknown (between 60 and 68%) in the anaerobic granules within this study, meaning that anaerobic processes remain to a large extent a black box of diversified and complex populations. On the archaeal side, the three sludges seem to have the acetoclastic and hydrogenophilic (including formate consumers) populations well balanced, with the genus *Methanosaeta* and the classes *Methanobacteriales* or *Methanomicrobiales* amply present in all sludges. The genera *Methanolinea* and *Methanomethylovorans* were absent in the Tembec and Lassonde sludges, respectively. No *Methanosarcina* has been found in any sludge studied.

**Table 2:** Relative abundance of bacterial and archaeal genera for each sludge

Sludge origin	Agropur (%)	Lassonde (%)	Tembec (%)
Bacteria			
*Desulfoglaeba*	18	3	2
*Mesotoga*	8	3	0
*Propioniciclava*	0	7	7
*Syntrophobacter*	5	1	0
*Propionibacterium*	0	4	1
*Desulfovibrio*	0	1	4
*Treponema*	3	3	4
*Aminivibrio*	1	2	4
*Desulfomicrobium*	0	2	3
*Syntrophorhabdus*	3	1	2
*Smithella*	0	2	1
*Olsenella*	0	0	2
*Alkalibaculum*	0	0	2
*Propionivibrio*	0	1	1
*Brooklawnia*	0	1	0
*Syntrophobotulus*	0	0	1
*Thermovirga*	0	0	1
*Sporobacter*	0	0	1
*Gaiella*	1	0	0
*Cloacibacillus*	0	0	1
*Propionicimonas*	0	1	0
*Flavonifractor*	0	1	0
*Syntrophus*	0	0	1
*Aminiphilus*	0	0	1
*Streptomyces*	0	1	0
*Petrimonas*	0	0	1
*Pelolinea*	0	0	0
Other or unknown	60	68	66
Archaea			
*Methanosaeta*	57	36	48
*Methanobacterium*	9	47	23
*Methanolinea*	25	6	0
*Methanomethylovorans*	3	0	8
*Methanomassiliicoccus*	1	1	2
Other or unknown	6	10	18

### EPS

A variety of physical and chemical methods have been proposed for EPS extraction, with variable results in terms of extraction yield of the extract (D’Abzac et al. 2010; Pellicer-Nàcher et al. 2013; Sheng et al. 2010). In our study, it was a physical extraction method that was chosen, since this is what was recommended in other studies on sludge proteome (Zhang et al. 2015; Park et al. 2008; Zhu et al. 2015). Although the yield differs according to the physical method used, the extracts show very similar IR spectra (Comte et al. 2006, D’Abzac et al. 2010), which guarantees their similarity. Glucose-6-phosphate dehydrogenase (G6PDH) activity was measured on EPS extract to evaluate the cell lysis during the sonication (Monique et al. 2008). No G6PDH activity was detected in extracts as shown in Table 3, while phase-contrast microscopy clearly showed alive cells after 8 min of sonication (Additional file 1: Figure S3). Composition of EPS extracted from three different anaerobic sludges is listed in Table 4. The EPS represented 31%, 20% and 14% of the dry weight (DW) of Agropur, Lassonde and Tembec sludge, respectively. The EPS compound with the smallest fraction was nucleic acids, followed by carbohydrates, while the protein fraction always represented the main compound, and HS, the second most important EPS constituent, for all sludges. Proteins and HS represent by consequence the core of this matrix where cells are embedded. The PN/PS ratio was 3, 3 and as high as 6 for Lassonde, Agropur and Tembec sludge, respectively.

**Table 3:** Cell lysis detection assays

	Negative control (buffer)	Positive controls (1.12 G6PDH units)	Positive controls incubate with samples	All samples
Activity slope (unit of absorbance (430 nm) per second)	0.0000 ± 0.0000	0.0168 ± 0.0010	0.0168 ± 0.0003	0.0000 ± 0.0000

Positive controls contained 1.12 units of glucose-6-phosphate dehydrogenase (G6PDH) in buffer. Positive controls incubated with cells samples were to test inhibitors presence in samples

**Table 4:** Compound classes of EPS extracted

Sludge origin	Agropur	Lassonde	Tembec
EPS/sludge [mg/g, DW]	311 ± 22	195 ± 6	143 ± 3
EPS/sludge [mg/g, VDW]	310 ± 33	151 ± 7	132 ± 3
Proteins EPS/sludge (mg/g, VDW)	124 ± 13	46 ± 17	66 ± 4
Humic substances EPS/sludge (mg/g, VDW)	44 ± 6	36 ± 6	47 ± 4
Carbohydrate EPS/sludge (mg/g, VDW)	37 ± 11	16 ± 1	11 ± 1
Nucleic acids EPS/sludge (mg/g, VDW)	16 ± 1	6 ± 1	6 ± 0
Protein/carbohydrate ratio	3	3	6

### Proteins in EPS

Proteins precipitation was done with EPS extracts prior separation on gels as shown in Fig. 1. The migration patterns were slightly different from one sludge to the other, but there were still bands of the same molecular weight in the 3 sludges. Proteins were ranging 26-89 kDa. Those results are consistent with previous studies (Zhu et al. 2015) where a thermal EPS extraction was used. After 3,3′,5,5′-tetramethylbenzidine (TMBZ) staining (Jensen et al. 2010), no metalloprotein (such as cytochromes) were detected on gels. The most representative bands on SDS-PAGE gels were cut and analyzed by HPLC-tandem mass spectrometry for protein identification. Additional file 1: Table S1 presents significant proteins that were identified. Four proteins were found in every sludge tested: the S-layer protein, the CO-methylating acetyl-CoA synthase complex (CODH/AC synthase), an ABC transporter substrate-binding protein and the coenzyme-B sulfoethylthiotransferase, also known as methyl-coenzyme M reductase (MCR). A total of 45 proteins from more than 50 species of archaea and bacteria have been identified (Table 5). *Methanosaeta concilii* is by far the microorganism that has been the most represented by this overall proteomic search. The Agropur sludge (cheese factory) was the less diversified sludge with only 31 positive hits and 17 proteins found, instead of 52 and 64 hits or 33 and 39 proteins found, for the Lassonde and Tembec sludge, respectively. It should also be mentioned that many sequenced peptides were not associated with any known organism or known protein, as already highlighted with the bacterial HTS results.

**Table 5:** Identified proteins and microbial species that produce them, in the EPS of the three tested sludges

Description	Species	Sludge
4-Hydroxy-tetrahydrodipicolinate synthase	*Methanosaeta concilii*	L T
5,10-Methylenetetrahydromethanopterin reductase	*Methanobacterium formicicum*	T
ABC transporter substrate-binding protein	*Methanosaeta concilii*	T
	*Candidatus Vecturithrix granuli*	A
	*Symbiobacterium thermophilum*	L
	*Flexilinea flocculi*	L
Acetate-CoA ligase	*Methanosaeta concilii*	A
Acetyl-CoA decarbonylase/synthase complex	*Methanosarcina mazei*	T
Acetyl-CoA synthase	*Methanosaeta concilii*	T
Adenylyl-sulfate reductase	*Candidatus Rokubacteria bacterium*	T
ATP-utilizing enzymes of ATP-grasp superfamily (probably carboligase)	*Methanobacterium*	T
Benzaldehyde dehydrogenase II	*Mycobacterium abscessus*	T
C-5 cytosine-specific DNA methylase family protein cellulase	*Clostridioides difficile*	T
	*Methanosaeta*	T
CO dehydrogenase/CO-methylating acetyl-CoA synthase complex	*Methanosaeta concilii*	A L T
Coenzyme-B sulfoethylthiotransferase (also known as methyl-coenzyme M reductase)	*Methanobacterium flexile*	L T
	*Methanobacterium formicicum*	L T
	*Methanobacterium paludis*	L T
	*Methanobrevibacter filiformis*	L T
	*Methanobrevibacter smithii*	L
	*Methanoculleus*	L
	*Methanolinea tarda*	A
	*Methanomethylovorans hollandica*	T
	*Methanoregula formica*	A
	*Methanosaeta concilii*	A L T
	*Methanosaeta harundinacea*	A
	*Methanosaeta thermophila PT*	T
	*Methanospirillum hungatei*	T
	*Methanothermobacter*	L T
Endonuclease/Exonuclease/phosphatase family protein	*Desulfobacterium vacuolatum*	T
Extracellular solute-binding protein family 1	*Candidatus Vecturithrix granuli*	A
FAD-binding oxidoreductase	*Dietzia*	T
Fasciclin domain protein	*Methanosaeta concilii*	T
Formate dehydrogenase	*Methanolinea tarda*	A
	*Pseudodesulfovibrio indicus*	A
Formate dehydrogenase-N	*Syntrophobacter fumaroxidans*	A
Glyceraldehyde 3-phosphate dehydrogenase	*Xenoturbella bocki*	L
	*Actinomyces cardiffensis*	L
	*Candidatus Synechococcus spongiarum*	L
	*Phytophthora nicotianae*	L
	*Microbacterium*	L T
	*Arsenicicoccus bolidensis*	L
	*Cutibacterium granulosum*	L
	*Demequina aurantiaca*	L
	*Elusimicrobia bacterium*	L
	*Planctomycetes bacterium*	T
	*Propionibacterium namnetense*	L
	*Saccharothrix espanaensis*	L
	*Serinicoccus profundi*	L
	*Tessaracoccus lapidicaptus*	L
Glycerol kinase	*Methylacidiphilum fumariolicum*	T
	*Spirosoma*	T
Ketol-acid reductoisomerase	*Methanosaeta concilii*	T
ll-diaminopimelate aminotransferase	*Methanosaeta concilii*	T
Manganese-dependent inorganic pyrophosphatase	*Methanosaeta concilii*	T
Methanol-cobalamin methyltransferase	*Methanomethylovorans hollandica*	T
Methanol-corrinoid methyltransferase	*Methanolobus profundi*	T
Methylmalonyl-CoA mutase	*Bacterium*	T
Mit domain-containing protein 1	*Ascaris suum*	L
Molecular chaperone DnaK	*Verrucomicrobia bacterium*	T
N5, N10-methylene tetrahydromethanopterin dehydrogenase (coenzyme F420-dependent)	*Methanococcus jannaschii*	T
Peptidase M42	*Methanosaeta concilii*	L
Peptidylprolyl isomerase	*Microbacterium*	L
Periplasmic-binding protein	*Methanosaeta concilii*	T
Phosphate-binding protein	*Methanosaeta concilii*	T
Phosphoglycerate kinase	*Propionibacterium*	L
Pyridoxal phosphate-dependent aminotransferase	*Mycobacterium palustre*	A
Radical SAM protein	*Clostridium cellulovorans*	L
Recombinase family protein	*Rhodobacter capsulatus*	A
S-layer protein	*Methanosaeta concilii*	A L T
	*Methanosaeta harundinacea*	A
Thioesterase	*Leptolyngbya*	A
TIGR03759 family integrating conjugative element protein	*Pseudoxanthomonas spadix*	A
Transketolase domain-containing protein	*Cephalotus follicularis*	L
tRNA delta(2)-isopentenylpyrophosphate transferase	*Ochrobactrum intermedium*	T
Uncharacterized membrane protein SpoIIM, required for sporulation	*Prevotella jejuni*	L
V-type H^+^-transporting ATPase	*Methanosaeta*	T

## Discussion

The EPS represent an important proportion of the granule studied, with from 14% to 31% by dry weight. Proteins were the main constituents. MS results show that most of the proteins identified were related to catalytic activities. Shotgun proteomic analysis of the cation exchange resin extracted EPS fraction from anaerobic sludge had reached the same conclusion (Zhang et al. 2015). This predominance of catabolic enzymes shows that the EPS matrix has the capacity for extracellular catabolic reactions, which represents a competitive advantage for anaerobic granules. After proteins, HS represent the second larger constituents of EPS. The HS of EPS could come from the biomass degradation or/and from polycondensation of relatively small molecules released during biomass decay, but also from synthesis by the microorganisms themselves (Claus et al. 1999). Their role in the microbial aggregates has not yet been clearly explained. HS in anaerobic environment may act as a reducing agent for electron transfer (Klüpfel et al. 2014; Roden et al. 2010; Voordeckers et al. 2010) and as such, promote direct interspecies electron transfer (DIET). As proteins extracted were mostly related to metabolism, HS are then the next candidate to play the structural role expected by EPS.

The variation in genera from one sludge to another is probably mainly due to the difference in the substrates composing the wastewater treated by the sludge, which can be preferentially consumed by one genus rather than another. *Methanosaeta concilii* is the microorganism for which the most protein hits were obtained regardless of the sludge origin. This archaeon has been reported as having a key role in the granulation (Hulshoff Pol et al. 2004). In most of granulation theories, *Methanosaeta* is largely associated with the first step of granulation, because it produces long filaments. S-Layer duplication domain protein from *Methanosaeta concilii* was found in the three protein extracts. S-Layer duplication domain protein is one of the primary proteins of the S-layer in archaea (Fagan and Fairweather 2014; Sara and Sleytr 2000). The archaeal S-layer proteins are known to have a structural role and to be involved in cell adhesion and population cohesion (De Vrieze et al. 2012). S-Layer proteins are typically highly glycosylated, leading to the formation of bonds between polysaccharides and proteinic EPS fractions (Sleytr and Beveridge 1999). The MCR and CODH/acetyl-CoA (CODH/AC) synthase enzymes were the other major proteins found in all sludges. MCR is an enzyme responsible for the last step of methanogenesis, combining the methyl group of coenzyme M with hydrogen from the coenzyme B to form methane (Balch et al. 1979). The CODH/AC synthase works primarily through the Wood–Ljungdahl pathway which converts CO_2_ to acetyl-CoA (Ragsdale and Pierce 2008). Archaeal methanogens, particularly *Methanosaeta*, largely predominate in the innermost layer of the granule (MacLeod et al. 1990; Sekiguchi et al. 1999). It is therefore expected that most dead archaea are also located in the granule centre, where they can accumulate, since they are confined in that remote zone of the EPS matrix and that they would be released in abundance during the sonication extraction. In this respect, Agropur granules typically were of light grey colour, indicating the lesser importance of such a usually dark methanogenic core; this would explain the lowest number of archaeal enzyme found in the EPS of Agropur granules.

The MCR and CODH/AC synthase enzymes are extremely sensitive to oxygen. When oxidized they are deactivated and could not be regenerated (Cedervall et al. 2010). Either the cell decomposes the deactivated enzymes for recycling, or simply excretes them, as recycling could cost too much energy. The presence of a large amount of MCR and CODH/AC synthase in EPS could then be a marker of oxygen stress undergone by methanogens. Small oxygen levels added in anaerobic reactors do not affect the methane production, however long-term exposure to O_2_ leads to smaller granule sizes in the reactors (Stephenson et al. 1999).

Among the other identified proteins, the glyceraldehyde 3-phosphate dehydrogenase (GAPDH) was largely found in Lassonde sludge and originating from many bacterial strains (Table 5). It actually represents the principal hit for bacterial strains overall the three sludges. GAPDH is an essential enzyme during glycolysis but it also has several other roles. It has been detected at the surface of several prokaryotes (Oliveira et al. 2012; Pancholi and Chhatwal 2003) as an adhesion and binding protein (Brassard et al. 2004). It binds albumin and several other mammalian proteins (Jin et al. 2005) and it is now considered as a virulence factor for some bacterial strains (Seidler 2013). GAPDH is required for EPS production in *Xanthomonas* proteobacteria (Lu et al. 2009). As a protein with multiple roles, GAPDH could play a role in granulation and in maintaining the granule integrity. Fasciclin domain proteins also have an important role in cell adhesion (Moody and Williamson 2013) and have been found expressed by *Methanosaeta concilii* in Tembec sludge.

Bacterial formate dehydrogenase (FDH) was identified from Agropur sludge proteins. This FDH is associated with molybdenum (Mo) or tungsten (W) and is NAD-independent. This enzyme catalyzes the reduction of CO_2_ using reducing equivalents coming from the upstream degradation of the primary substrate. FDH could also oxidize formate to produce CO_2_ and reducing equivalents. The beta subunit has a transmembrane domain that allows the conduction of electrons within the protein. This enzyme is probably a key enzyme to regulate the formate catabolism in granules as it can both produce and consume formate and in this way ensure that thermodynamic conditions are favourable during the degradation of organic matter (Crable et al. 2011). As this enzyme is NAD-independent and possesses its own catalyzer, it could be extracellularly active, expanding the regulation of formate in the extracellular environment. As for hydrogen, increasing formate concentration rapidly prevents its production, limiting the oxidation of substrates. This ability to excrete FDH in the extracellular environment would therefore be a considerable advantage for bacteria, since it could reduce the concentration of formate faster than its diffusion and intracellular consumption by methanogens. In such a scenario, FDH would externally produce reducing equivalents and CO_2_. The reducing equivalents could then be used by methanogens via DIET to reduce CO_2_ to methane, since *Methanosaeta* and *Methanosarcina*, in addition to acetate, are able to consume free electrons via DIET (Rotaru et al. 2014a, b). In Agropur sludge, *Methanosaeta* was found to represent 57% of the archaeal genera identified (Table 2). Perhaps and contrary to what was acknowledged in the past, *Methanosaeta* could be a major player in non-acetoclastic methanogenesis in granular sludge (Smith and Ingram-Smith 2007). Another bacterial FDH, the nitrate-inducible FDH or FDH-N, was found in Agropur sludge. This FDH-N plays a major role in the respiration of nitrate. This is likely related to the fact that Agropur sludge treats proteins-containing wastewater, and therefore, that the nitrogen cycle is more prominent than in Lassonde and Tembec sludge. For the same reason, cellulase has been found specifically in Tembec EPS, which is in direct links with typical paper mill wastewater substrates. The role of the extracellular enzyme is likely more instrumental in a confined environment such as the innermost region of granules than in the open medium of a free suspension. This likely adds to the competitive advantages of biofilm as compared to planktonic growth.

As previously mentioned and shown here, substrate impacts bacterial populations present in granules from different origins (Table 2). It also impacts the diversity, which was much lower in Agropur sludge than in Tembec sludge, for example. Archaeal populations also vary, but the balance between acetoclastic and hydrogenophilic methanogens seems to be fairly stable despite the dissimilarity of treated wastewater. The origin of the sludge also has an impact on the characteristics of the EPS. Although approximately the same amount of HS was found in every sludge, protein and carbohydrate fractions varied more than 3 times from one sludge to another (Table 4). With regard to the different proteins identified and the microbial species that produce them, since very few structural proteins have been detected, it is difficult to relate the role of the different species present to the structural function of the EPS. However, it seems that EPS could be an important site for enzymatic reactions, since many catabolic enzymes have been found there. Further analysis is needed to understand the role of these enzymes in the ecology of granules, as very few studies have been published on the protein fraction of EPS in granules. Some studies have characterized the protein fraction of EPS only in aerobic sludge flocs and granules. The current study improved the quantification and contributed to the characterization of the EPS protein fraction for anaerobic granules expressly.

## Additional file

**Additional file 1: Figure S1.** Measured protein concentration. **Figure S2.** Measured concentration of humic substances. **Figure S3.** Microphotographs of sonicated granules, showing alive cells. **Table S1.** Analysis of the protein fraction of the extracellular polymeric substances by tandem mass spectrometry.
