# Supplementary material for: Characterization of the protein fraction of the extracellular polymeric substances of three anaerobic granular sludges
Source: AMB Express. 2019 Feb 7;9:23. doi: 10.1186/s13568-019-0746-0 (PMC6367495; doi:10.1186/s13568-019-0746-0)
Supplement: Supplementary file 1 — Additional file 1: Figure S1. Measured protein concentration. Figure S2. Measured concentration of humic substances. Figure S3. Microphotographs of sonicated granules, showing alive cells. Table S1. Analysis of the protein fraction of the extracellular polymeric substances by tandem mass spectrometry. [file 13568_2019_746_MOESM1_ESM.pdf]

AMB Express

Characterization of the protein fraction of the extracellular polymeric substances of three anaerobic granular sludges

Charles-David Dubé,[1,2] and Serge R. Guiot, [1,2,*]

[1] Anaerobic Bioprocesses Group, Energy, Mining and Environment Research Center, National Research Council Canada, Montreal, Canada
[2] Department of Microbiology, Infectiology and Immunology, Université de Montréal, Montreal, Canada

* For correspondence. E-mail serge.guiot@umontreal.ca; Tel +1 438 4966181; Fax +1 514 4966265

Additional file 1

Update of the Frølund's colorimetric method for the determination of mixed proteins and humic substances

Introduction

In 1995, Frølund's team proposed a modified method of Lowry's procedure to determine proteins and humic substances (HS) (Frølund et al. 1995). As the Folin-Ciocalteu's phenol reagent (FCPR) is used for colorimetric determination of both proteins and HS (Box 1983; Lowry et al. 1951), they estimated which part of the color development was due to protein versus HS in samples that contain both. With $CuSO_4$, the total absorbance is equal to the sum of the absorbances from proteins and HS. Without $CuSO_4$, the color development (blind absorbance) is due to HS, in addition to chromogenic amino acids. They observed that HS were responsible for a decrease of the color developed by proteins to 20% but there was no decrease for humic acids, in the absence of $CuSO_4$. The mutual interference of proteins and humic compounds was addressed as following:

$$A_{total} = A_{protein} + A_{humic} \qquad (1)$$

$$A_{protein} = 1.25\,(A_{total} - A_{blind}) \qquad (2)$$

$$A_{humic} = A_{blind} - 1/5\,A_{protein} \qquad (3)$$

where A_{total} is the total absorbance at 750 nm (A_{750}) with $CuSO_4$, A_{blind} is the total A_{750} without $CuSO_4$, A_{humic} is the A_{750} due to humic compounds, and $A_{protein}$ is the A_{750} due to proteins.

In the particular case of exopolymeric substances (EPS) extracted from mixed anaerobic biofilms, those equations yielded results particularly high in HS and low in proteins, compared to the literature. Following further investigation, we propose here to update the Frølund's method for

the determination of proteins and humic substances when they are both present in the same sample.

Material and methods

Bovine serum albumin (BSA) (Sigma-Aldrich #A7030, MilliporeSigma Canada Co., Oakville, Ontario) and humic acid (HA) (Sigma-Aldrich #53680) were used as the standards for measurement of proteins and humic compounds, respectively. A dilution series of calibration standards in water were prepared, either with HA, or BSA, to give concentrations of 0 to 1 mg/mL. A few drops of concentrated NaOH were used to dissolve HA in water. Solution A is freshly made daily with 50 mL of solution B and 1 mL of solution C. Solution B contains 2% Na_2CO_3 in 0.1M NaOH. Solution C contains 1% sodium tartrate dihydrate with or without 0.5% $CuSO_4$. A few drops of concentrated H_2SO_4 were used to dissolve $CuSO_4$. Solution D contains FCPR (purchased from Sigma-Aldrich, #47641) diluted with an equal volume of HCl 2M. Each mL of sample is mixed with 5 mL of solution A. Thereafter within maximum 10 minutes, 0.5 mL of solution D is added and immediately mixed. Absorbance is read at 750 nm after between minimum 30 minutes and maximum 1 hour using a spectrophotometer (DR3900, Hach, London, Ontario). Total absorbance (A_{total}) was measured in presence of $CuSO_4$ while blind absorbance (A_{blind}) was measured with $CuSO_4$ omitted, using the series of standards with known BSA or HA concentrations (C_{BSA} or C_{HA}, in mg/mL), to yield the calibration factors from the calibration plot slope, either F_{BSA} (i.e. $\Delta C_{BSA}/\Delta A_{total}$) or F_{HA} (i.e. $\Delta C_{HA}/\Delta A_{total}$).

Results

Mixed solutions of HA and BSA were prepared with various combined concentrations of HA and BSA from 0 to 1 mg/mL for each. Total absorbance (A_{total}) and blind absorbance (A_{blind}) were measured on all samples, and the concentrations of proteins and humic substances were estimated using the formulas (4) and (5) below, to be confronted with their nominal values (concentrations added) in order to test the adequacy of the Frølund's equations (2) and (3) above.

$$C_{protein} = A_{protein} F_{BSA} \quad (4)$$

$$C_{humic} = A_{humic} F_{HA} \quad (5)$$

where $C_{protein}$ and C_{humic} are the concentrations (mg/mL) in proteins and humic substances, respectively, and $A_{protein}$ and A_{humic} are given by the equations (2) and (3), respectively.

Figure 1-A diplays the plots of the protein concentration measured using the Frølund's equations as above described, as a function of the nominal or added concentration of proteins, with no HA added and with different concentrations of HA added. That is, each dot corresponds to the average measured protein concentration of several samples, all with the same protein concentration added, while humic acid concentrations ranged from 0 to 1 mg/mL. We observed that the discrepancy between the measured BSA concentration as estimated according to Frølund and the expected concentrations is inversely proportional the concentration of the HA that the sample contains (not shown). The presence of HA in the assay clearly lowers the detection of BSA. In presence of 1 mg/mL of HA, the BSA concentration is underestimated by almost half. Even in absence of HA, BSA concentrations remain underestimated by 30% when calculated

with equations (4) and (2).

Similarly, Figure 2-A diplays the plots of the HA concentration measured using the Frølund's equations as above described, as a function of the nominal or added concentration of HA when proteins are absent and with different protein concentrations. That is, each dot corresponds to the average measured HA concentration of several samples, all with the same HA concentration added, while BSA concentrations ranged from 0 to 1 mg/mL. As with the results obtained for protein measurements, the change for the HA estimates using equations (5) and (3) is directly related to the added BSA concentration (not shown). When there is no BSA, the HA estimated value coincides with the expected value. In contrast, the addition of 1 mg/mL of BSA in the samples overestimates the estimated values by 0.1 mg/mL, for expected HA concentrations near 1 mg/mL, and by 0.3 mg/mL, when HA concentration tends to zero.

This means that chromogenic amino acids represent a higher color development in the absence of $CuSO_4$ than previously described. We estimated that this color development was higher by 40% instead of 20% (equation (8) instead of equation (3)). A ration *R* (equation 6) is also introduced in equation (2) to offset the underestimation of protein fraction when HA fraction tends to be high (Figure 1-A), and the coefficient of the total absorbance in the same equation is reduced accordingly (equation 7). The use of equations (6), (7) and (8) as developed below makes it possible to reconcile the experimental and expected values as shown in Figure 1-B and 2-B, respectively.

$$R = A_{blind} / A_{total} \qquad (6)$$

$$A_{protein} = 1.15\ A_{total} - R\ A_{blind} \qquad (7)$$

$$A_{humic} = A_{blind} - 0.4\ A_{protein} \qquad (8)$$

References

Box JD (1983) Investigation of the Folin-Ciocalteau phenol reagent for the determination of polyphenolic substances in natural waters. Water Res 17:511-525. https://doi.org/10.1016/0043-1354(83)90111-2

Frølund B, Griebe T, Nielsen PH (1995) Enzymatic activity in the activated-sludge floc matrix. Appl Microbiol Biotechnol 43(4):755-61. https://doi.org/10.1007/s002530050481

Lowry OH, Rosenburg NJ, Farr AL, Randall RJ (1951) Protein measurement with Folin reagent. J Biol Chem 103:265-275.

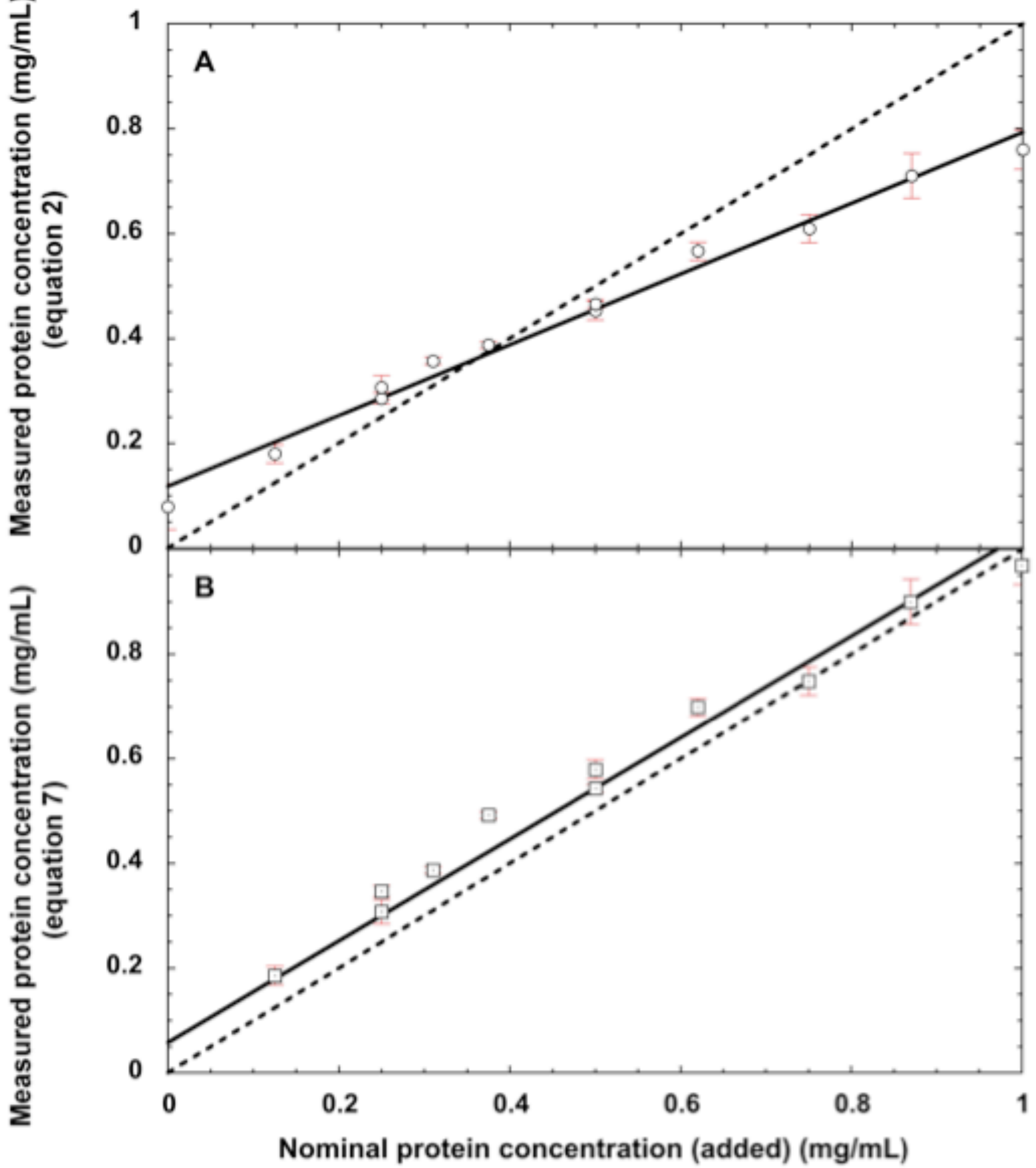

Fig. S1. Measured protein concentration (A, using the Frølund's equation (2); B, using the newly developed equation (7)), as a function of the nominal (added) protein concentration. Each dot corresponds to the average measured protein concentration of several samples, all with the same protein concentration added, while humic acid concentrations were varying from 0 to 1 mg/mL.

------- Expected concentration (measured = nominal values)

_____ Linear regression curve: (A) $C_{measured} = 0.112 + 0.676\ C_{nominal}$ ($R = 0.994$); (B) $C_{measured} = 0.055 + 0.971\ C_{nominal}$ ($R = 0.984$)

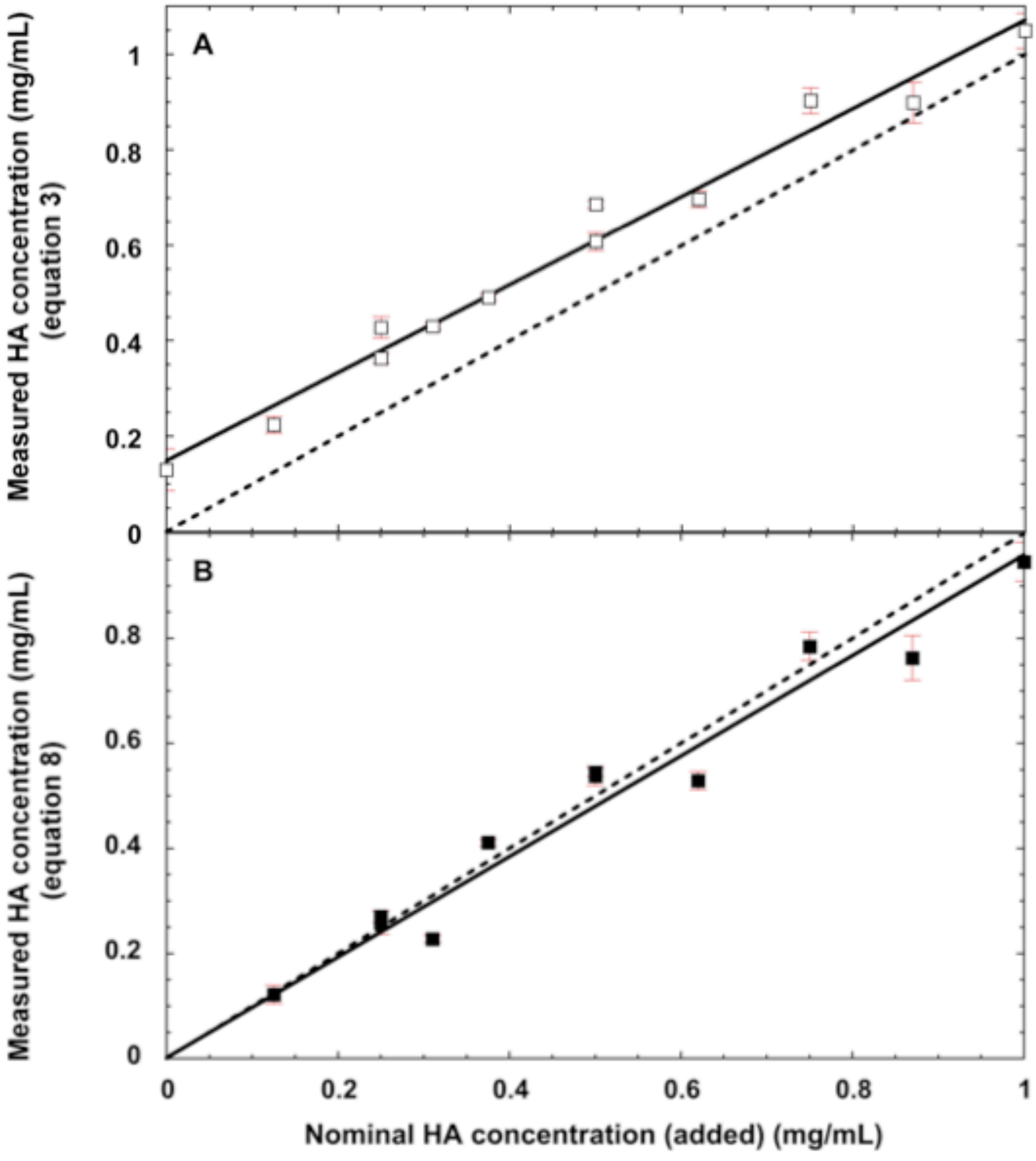

Fig. S2. Measured concentration of humic substances (HA) (A, using the Frølund's equation (3); B, using the newly developed equation (8)), as a function of the nominal (added) HA concentration. Each dot corresponds to the average measured HA concentration of several samples, all with the same HA concentration added, while BSA concentrations were varying from 0 to 1 mg/mL.

------- Expected concentration (measured = nominal values)

_____ Linear regression curve: (A) $C_{measured} = 0.15 + 0.92\ C_{nominal}$ (R = 0.99); (B) $C_{measured} = 0.0025 + 0.96\ C_{nominal}$ (R = 0.985)

Fig S3. Microphotographs of sonicated granules, showing alive cells.

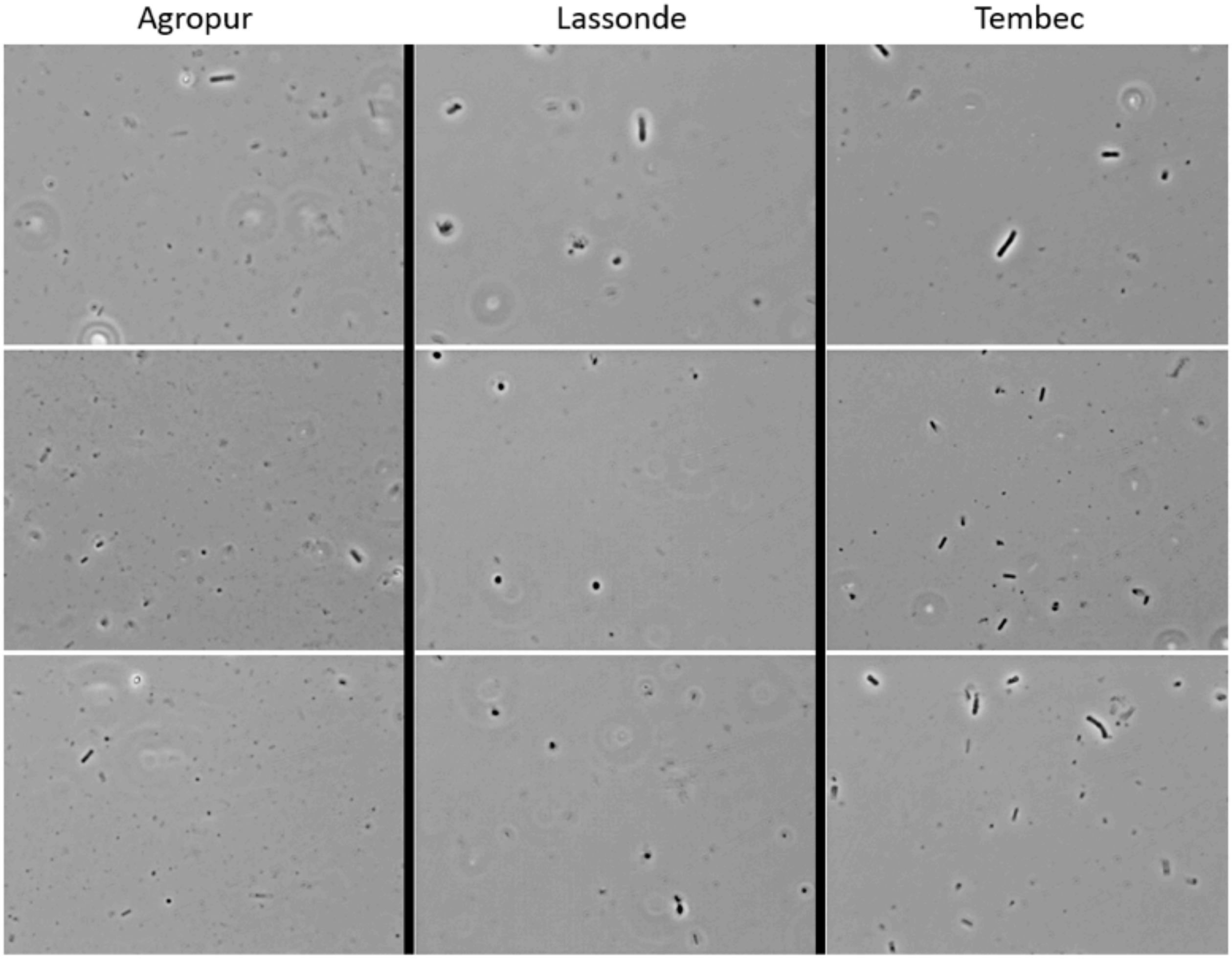

Phase-contrast microscopy (Laborlux S, Leitz, Germany; magnification 1000X). Cells after 8 minutes of sonication, dilution 1/100 in buffer.

Table S1. Analysis of the protein fraction of the extracellular polymeric substances by tandem mass spectrometry. Proteins identified for the three anaerobic sludges, and microbial origin.

Accession	Score	Mass	Num. of matches	Num. of sequences	Description	Species
**AGROPUR SLUDGE**						
AMK11930.1	51	91	1	1	formate dehydrogenase	*Pseudodesulfovibrio indicus*
WP_011696912.1	70	89	2	2	formate dehydrogenase-N subunit alpha	*Syntrophobacter fumaroxidans*
OYV08313.1	104	80	3	2	S-layer-related duplication domain-containing protein	*Methanosaeta* sp. ASP1-1
WP_013718926.1	190	77	6	4	S-layer protein	*Methanosaeta concilii*
WP_013718926.1	114	77	4	4	S-layer protein	*Methanosaeta concilii*
WP_013718926.1	63	77	3	3	S-layer protein	*Methanosaeta concilii*
WP_042688872.1	57	76	1	1	formate dehydrogenase subunit alpha	*Methanolinea tarda*
WP_013718459.1	57	74	1	1	acetate--CoA ligase	*Methanosaeta concilii*
GAK56414.1	58	64	1	1	ABC transporter, substrate binding protein	*Candidatus vecturithrix granuli*
WP_081035769.1	69	62	1	1	recombinase family protein	*Rhodobacter capsulatus*
WP_014586065.1	85	61	3	2	coenzyme-B sulfoethylthiotransferase subunit alpha	*Methanosaeta harundinacea*
WP_013718622.1	119	61	6	4	coenzyme-B sulfoethylthiotransferase subunit alpha	*Methanosaeta concilii*
WP_013718622.1	116	61	3	3	coenzyme-B sulfoethylthiotransferase subunit alpha	*Methanosaeta concilii*
WP_013718622.1	96	61	3	3	coenzyme-B sulfoethylthiotransferase subunit alpha	*Methanosaeta concilii*
WP_013719066.1	75	52	2	2	CO dehydrogenase/CO-methylating acetyl-CoA synthase complex subunit beta	*Methanosaeta concilii*
KUK43246.1	55	50	2	2	Methyl-coenzyme M reductase, alpha subunit, partial	*Methanosaeta harundinacea*
GAK59571.1	77	49	1	1	extracellular solute-binding protein family 1	*Candidatus Vecturithrix granuli*
GAK59735.1	73	46	2	2	extracellular solute-binding protein family 1	*Candidatus Vecturithrix granuli*
WP_015286559.1	111	46	3	1	coenzyme-B sulfoethylthiotransferase subunit beta	*Methanoregula formicica*
KUK43225.1	268	46	11	6	Methyl-coenzyme M reductase, beta subunit	*Methanosaeta harundinacea*
OYV09330.1	407	45	18	10	methyl-coenzyme M reductase beta subunit	*Methanosaeta* sp. NSP1
OYV09330.1	75	45	1	1	methyl-coenzyme M reductase beta subunit	*Methanosaeta* sp. NSP1
WP_013718625.1	593	45	22	14	coenzyme-B sulfoethylthiotransferase subunit beta	*Methanosaeta concilii*
WP_013718625.1	100	45	1	1	coenzyme-B sulfoethylthiotransferase subunit beta	*Methanosaeta concilii*
BAL72752.1	75	43	1	1	methyl-coenzyme M reductase alpha subunit, partial	*Methanolinea tarda*
BAL72752.1	73	43	1	1	methyl-coenzyme M reductase alpha subunit, partial	*Methanolinea tarda*
WP_085077459.1	56	36	1	1	pyridoxal phosphate-dependent aminotransferase	*Mycobacterium palustre*
KUK44334.1	54	33	1	1	S-layer-related duplication domain protein, partial	*Methanosaeta harundinacea*
WP_007314360.1	57	29	1	1	coenzyme-B sulfoethylthiotransferase subunit gamma	*Methanolinea tarda*
WP_014159660.1	48	27	1	1	TIGR03759 family integrating conjugative element protein	*Pseudoxanthomonas spadix*
WP_008313207.1	54	22	2	1	thioesterase	*Leptolyngbya* sp. PCC 6406
**LASSONDE SLUDGE**						
GAV78649.1	51	80	1	1	Transketolase domain-containing protein	*Cephalotus follicularis*
WP_013718926.1	291	77	13	8	S-layer protein	*Methanosaeta concilii*
WP_013718926.1	268	77	9	7	S-layer protein	*Methanosaeta concilii*
CVK33165.1	81	63	1	1	Methyl-coenzyme M reductase I subunit alpha	*Methanoculleus* sp. MAB1
WP_013718622.1	157	61	6	6	coenzyme-B sulfoethylthiotransferase subunit alpha	*Methanosaeta concilii*
WP_004031277.1	433	61	13	7	coenzyme-B sulfoethylthiotransferase subunit alpha	*Methanobacterium formicicum*
EKQ53862.1	158	61	4	4	methyl-coenzyme M reductase, alpha subunit	*Methanobacterium* sp. Maddingley MBC34
WP_023992801.1	371	61	12	7	coenzyme-B sulfoethylthiotransferase subunit alpha	*Methanobacterium* sp. MB1
WP_023992801.1	148	61	4	4	coenzyme-B sulfoethylthiotransferase subunit alpha	*Methanobacterium* sp. MB1
WP_013826564.1	81	60	2	2	coenzyme-B sulfoethylthiotransferase subunit alpha	*Methanobacterium paludis*
WP_013291967.1	48	56	1	1	radical SAM protein	*Clostridium cellulovorans*
WP_013719066.1	85	52	2	2	CO dehydrogenase/CO-methylating acetyl-CoA synthase complex subunit beta	*Methanosaeta concilii*
WP_013826568.1	188	47	5	3	coenzyme-B sulfoethylthiotransferase subunit beta	*Methanobacterium paludis*
WP_048072495.1	233	47	12	8	coenzyme-B sulfoethylthiotransferase subunit beta	*Methanobacterium formicicum*
WP_048072495.1	92	47	1	1	coenzyme-B sulfoethylthiotransferase subunit beta	*Methanobacterium formicicum*
WP_048072495.1	84	47	2	2	coenzyme-B sulfoethylthiotransferase subunit beta	*Methanobacterium formicicum*
WP_004032914.1	62	47	1	1	coenzyme-B sulfoethylthiotransferase subunit beta	*Methanobrevibacter smithii*
WP_004031273.1	205	47	11	8	coenzyme-B sulfoethylthiotransferase subunit beta	*Methanobacterium formicicum*
WP_066972983.1	123	47	2	2	coenzyme-B sulfoethylthiotransferase subunit beta	*Methanobrevibacter filiformis*

WP_066972983.1	57	47	1	1	coenzyme-B sulfoethylthiotransferase subunit beta	*Methanobrevibacter filiformis*
WP_013718625.1	74	45	3	3	coenzyme-B sulfoethylthiotransferase subunit beta	*Methanosaeta concilii*
EPH00329.1	71	42	1	1	phosphoglycerate kinase	*Propionibacterium* sp. oral taxon 192 str. F0372
ADM52196.1	131	41	3	3	methyl-coenzyme M reductase alpha subunit, partial	*Methanobacterium flexile*
ADM52196.1	88	41	1	1	methyl-coenzyme M reductase alpha subunit, partial	*Methanobacterium flexile*
ADM52196.1	84	41	2	2	methyl-coenzyme M reductase alpha subunit, partial	*Methanobacterium flexile*
OGS20011.1	110	38	5	2	type I glyceraldehyde-3-phosphate dehydrogenase	*Elusimicrobia bacterium* RIFOXYA2_FULL_39_19
WP_013720082.1	77	38	1	1	peptidase M42	*Methanosaeta concilii*
KKZ10447.1	66	37	1	1	glyceraldehyde-3-phosphate dehydrogenase	*Candidatus Synechococcus spongiarum* SP3
SCY12049.1	200	37	6	2	glyceraldehyde-3-phosphate dehydrogenase (NAD+)	*Microbacterium* sp. LKL04
SCY12049.1	116	37	2	2	glyceraldehyde-3-phosphate dehydrogenase (NAD+)	*Microbacterium* sp. LKL04
SCY12049.1	92	37	2	2	glyceraldehyde-3-phosphate dehydrogenase (NAD+)	*Microbacterium* sp. LKL04
SCY12049.1	66	37	1	1	glyceraldehyde-3-phosphate dehydrogenase (NAD+)	*Microbacterium* sp. LKL04
WP_062283483.1	71	37	1	1	BMP family ABC transporter substrate-binding protein	*Flexilinea flocculi*
ENO17529.1	197	36	8	4	glyceraldehyde-3-phosphate dehydrogenase	*Actinomyces cardiffensis* F0333
WP_002548822.1	162	36	7	3	type I glyceraldehyde-3-phosphate dehydrogenase	*Propionibacterium namnetense*
WP_021104351.1	141	36	6	2	type I glyceraldehyde-3-phosphate dehydrogenase	*Cutibacterium granulosum*
WP_015103632.1	85	36	2	2	type I glyceraldehyde-3-phosphate dehydrogenase	*Saccharothrix espanaensis*
WP_061965983.1	70	36	1	1	type I glyceraldehyde-3-phosphate dehydrogenase	*Demequina aurantiaca*
WP_029210506.1	90	36	1	1	type I glyceraldehyde-3-phosphate dehydrogenase	*Arsenicicoccus bolidensis*
WP_068752506.1	130	35	2	2	type I glyceraldehyde-3-phosphate dehydrogenase	*Tessaracoccus lapidicaptus*
WP_010148539.1	123	35	3	2	type I glyceraldehyde-3-phosphate dehydrogenase	*Serinicoccus profundi*
SNR67343.1	41	35	1	1	Uncharacterized membrane protein SpoIIM, required for sporulation	*Prevotella jejuni*
ABM97669.1	33	35	1	1	glyceraldehyde 3-phosphate dehydrogenase, partial	*Xenoturbella bocki*
OTA40626.1	74	32	1	1	branched-chain amino acid ABC transporter substrate-binding protein, partial	*Symbiobacterium thermophilum*
KUF91121.1	119	32	5	2	Glyceraldehyde-3-phosphate dehydrogenase	*Phytophthora nicotianae*
WP_013718654.1	58	31	2	2	4-hydroxy-tetrahydrodipicolinate synthase	*Methanosaeta concilii*
BAW31875.1	63	29	1	1	methyl-coenzyme M reductase I, subunit gamma	*Methanothermobacter* sp. MT-2
ERG83110.1	46	29	1	1	mit domain-containing protein 1	*Ascaris suum*
WP_004031276.1	103	28	4	3	coenzyme-B sulfoethylthiotransferase subunit gamma	*Methanobacterium formicicum*
ODT28037.1	71	23	1	1	type I glyceraldehyde-3-phosphate dehydrogenase, partial	*Microbacterium* sp. SCN 70-27
ODT28037.1	70	23	1	1	type I glyceraldehyde-3-phosphate dehydrogenase, partial	*Microbacterium* sp. SCN 70-27
WP_023953331.1	51	22	1	1	peptidylprolyl isomerase	*Microbacterium* sp. TS-1
**TEMBEC SLUDGE**						
AEB68019.1	219	88	7	6	CO dehydrogenase/acetyl-CoA synthase complex, alpha subunit	*Methanosaeta concilii* GP6
WP_013718926.1	95	77	2	2	S-layer protein	*Methanosaeta concilii*
WP_013718926.1	77	77	3	3	S-layer protein	*Methanosaeta concilii*
SMD10738.1	48	73	1	1	Endonuclease/Exonuclease/phosphatase family protein	*Desulfobacterium vacuolatum* DSM 3385
OGL04997.1	39	70	1	1	adenylyl-sulfate reductase subunit alpha	*Candidatus rokubacteria bacterium* RIFCSPLOWO2_02_FULL_68_19
PAW70078.1	46	68	1	1	molecular chaperone DnaK	*Verrucomicrobiae bacterium* Tous-C5FEB
WP_011449112.1	47	63	1	1	coenzyme-B sulfoethylthiotransferase subunit alpha	*Methanospirillum hungatei*
OAI44204.1	62	63	1	1	methylmalonyl-CoA mutase	*Bacterium* SCGC AG-212-C10
WP_013718622.1	351	61	14	10	coenzyme-B sulfoethylthiotransferase subunit alpha	*Methanosaeta concilii*
WP_013718622.1	60	61	1	1	coenzyme-B sulfoethylthiotransferase subunit alpha	*Methanosaeta concilii*
AEB68670.1	117	58	3	3	oligopeptide ABC transporter, solute-binding protein	*Methanosaeta concilii* GP6
WP_009059370.1	44	56	1	1	glycerol kinase	*Methylacidiphilum fumariolicum*
OJW77027.1	66	55	1	1	glycerol kinase	*Spirosoma* sp. 48-14
SHQ02298.1	86	54	1	1	benzaldehyde dehydrogenase II	*Mycobacterium abscessus subsp. abscessus*
WP_013719063.1	128	52	3	3	acetyl-CoA synthase subunit gamma	*Methanosaeta concilii*
WP_013719063.1	87	52	3	3	acetyl-CoA synthase subunit gamma	*Methanosaeta concilii*
WP_013719066.1	72	52	3	3	CO dehydrogenase/CO-methylating acetyl-CoA synthase complex subunit beta	*Methanosaeta concilii*
WP_015324191.1	98	51	2	2	methanol-cobalamin methyltransferase B subunit	*Methanomethylovorans hollandica*
WP_091936189.1	83	50	2	2	methanol--corrinoid methyltransferase	*Methanolobus profundi*
WP_013719695.1	76	50	1	1	ABC transporter substrate-binding protein	*Methanosaeta concilii*

WP_013719707.1	79	49	1	1	periplasmic-binding protein	*Methanosaeta concilii*
OYV09307.1	121	49	3	3	V-type H+-transporting ATPase subunit B	*Methanosaeta* sp. NSP1
WP_013719064.1	79	48	1	1	CO dehydrogenase/acetyl-CoA synthase subunit delta	*Methanosaeta concilii*
WP_013826568.1	75	47	2	1	coenzyme-B sulfoethylthiotransferase subunit beta	*Methanobacterium paludis*
WP_048072495.1	86	47	1	1	coenzyme-B sulfoethylthiotransferase subunit beta	*Methanobacterium formicicum*
WP_048072495.1	66	47	2	1	coenzyme-B sulfoethylthiotransferase subunit beta	*Methanobacterium formicicum*
WP_066972983.1	99	47	1	1	coenzyme-B sulfoethylthiotransferase subunit beta	*Methanobrevibacter filiformis*
WP_066972983.1	98	47	1	1	coenzyme-B sulfoethylthiotransferase subunit beta	*Methanobrevibacter filiformis*
WP_013719720.1	75	47	5	5	periplasmic-binding protein	*Methanosaeta concilii*
ABK14363.1	53	46	1	1	methyl-coenzyme M reductase, beta subunit	*Methanosaeta thermophila* PT
AEB68256.1	60	46	1	1	periplasmic binding protein	*Methanosaeta concilii* GP6
WP_015325024.1	82	46	1	1	coenzyme-B sulfoethylthiotransferase subunit beta	*Methanomethylovorans hollandica*
OYV09330.1	62	45	1	1	methyl-coenzyme M reductase beta subunit	*Methanosaeta* sp. NSP1
WP_013718625.1	411	45	14	8	coenzyme-B sulfoethylthiotransferase subunit beta	*Methanosaeta concilii*
WP_013718625.1	252	45	6	5	coenzyme-B sulfoethylthiotransferase subunit beta	*Methanosaeta concilii*
WP_013718625.1	108	45	3	3	coenzyme-B sulfoethylthiotransferase subunit beta	*Methanosaeta concilii*
WP_013718625.1	100	45	3	3	coenzyme-B sulfoethylthiotransferase subunit beta	*Methanosaeta concilii*
WP_013718625.1	81	45	3	3	coenzyme-B sulfoethylthiotransferase subunit beta	*Methanosaeta concilii*
WP_067718779.1	49	45	1	1	FAD-binding oxidoreductase	*Dietzia* sp. 111N12-1
WP_013718386.1	63	42	2	2	ABC transporter substrate-binding protein	*Methanosaeta concilii*
WP_013719904.1	62	42	1	1	LL-diaminopimelate aminotransferase	*Methanosaeta concilii*
BAL63107.1	93	42	2	2	methyl-coenzyme M reductase alpha subunit, partial	*Methanosaeta concilii*
BAL63107.1	53	42	1	1	methyl-coenzyme M reductase alpha subunit, partial	*Methanosaeta concilii*
ADM52196.1	69	41	2	2	methyl-coenzyme M reductase alpha subunit, partial	*Methanobacterium flexile*
EKQ54804.1	63	41	1	1	ATP-utilizing enzymes of ATP-grasp superfamily (probably carboligase)	*Methanobacterium* sp. Maddingley MBC34
WP_013719754.1	127	40	4	4	ABC transporter substrate-binding protein	*Methanosaeta concilii*
SCY12049.1	75	37	1	1	glyceraldehyde-3-phosphate dehydrogenase (NAD+)	*Microbacterium* sp. LKL04
WP_013718333.1	66	37	2	2	ketol-acid reductoisomerase	*Methanosaeta concilii*
pir||E64491	64	37	1	1	N5,N10-methylenetetrahydromethanopterin dehydrogenase (coenzyme F420-dependent)	*Methanococcus jannaschii*
OQY04731.1	57	36	1	1	type I glyceraldehyde-3-phosphate dehydrogenase	*Planctomycetales bacterium* 4572_13
KKG40403.1	75	34	1	1	acetyl-CoA decarbonylase/synthase complex subunit beta	*Methanosarcina mazei*
WP_013719528.1	124	34	2	2	manganese-dependent inorganic pyrophosphatase	*Methanosaeta concilii*
WP_004030775.1	89	33	2	2	5,10-methylenetetrahydromethanopterin reductase	*Methanobacterium formicicum*
AEB67029.1	87	33	1	1	fasciclin domain protein	*Methanosaeta concilii* GP6
AEB67029.1	70	33	1	1	fasciclin domain protein	*Methanosaeta concilii* GP6
WP_013718654.1	69	31	2	2	4-hydroxy-tetrahydrodipicolinate synthase	*Methanosaeta concilii*
WP_013719834.1	55	29	2	2	phosphate-binding protein	*Methanosaeta concilii*
BAW31875.1	46	29	1	1	methyl-coenzyme M reductase I, subunit gamma	*Methanothermobacter* sp. MT-2
WP_013718623.1	410	28	15	7	coenzyme-B sulfoethylthiotransferase subunit gamma	*Methanosaeta concilii*
WP_015325027.1	96	28	2	2	coenzyme-B sulfoethylthiotransferase subunit gamma	*Methanomethylovorans hollandica*
ELT48911.1	53	20	1	1	tRNA delta(2)-isopentenylpyrophosphate transferase	*Ochrobactrum intermedium* M86
OYV07818.1	86	16	1	1	cellulase, partial	*Methanosaeta* sp. ASP1-2
EQH17102.1	15	9	1	1	C-5 cytosine-specific DNA methylase family protein, partial	*Clostridioides difficile* DA00210
OYV10830.1	57	7	1	1	cellulase, partial	*Methanosaeta* sp. NSP1
